# The Extract of *Siegesbeckia orientalis* L. Reverses Pro-inflammatory Status of Microglia for Neuroprotection Following Ischemic Stroke in Mice

**DOI:** 10.2174/011570159X349127241214045611

**Published:** 2025-04-25

**Authors:** Liu Bowen, Wan Bingjie, Zhao Xinyue, Zhao Yonghua, Yu Hua, Jiang Xuhong, Zheng Yanrong, Xu Yun

**Affiliations:** 1 School of Pharmaceutical Sciences, The First Affiliated Hospital of Zhejiang Chinese Medical University (Zhejiang Provincial Hospital of Chinese Medicine), Zhejiang Chinese Medical University, Hangzhou, China;; 2 Department of Neurology, Suzhou Hospital, Xiyuan Hospital of China Academy of Chinese Medical Sciences (Suzhou TCM Hospital Affiliated to Nanjing University of Chinese Medicine), Suzhou, China;; 3 State Key Laboratory of Quality Research in Chinese Medicine, Institute of Chinese Medical Sciences, University of Macau, Macao, SAR 999078, China;; 4 Department of Neurology, Nanjing Drum Tower Hospital, Affiliated Hospital of Medical School, Nanjing University, Nanjing, China;; 5 State Key Laboratory of Pharmaceutical Biotechnology and Institute of Translational Medicine for Brain Critical Diseases, Nanjing University, Nanjing, China

**Keywords:** *Siegesbeckia orientalis* L., inflammation, microglia-neuron coupling, apoptosis, pyroptosis, ischemic stroke

## Abstract

**Background:**

*Siegesbeckia orientalis* L. (SO) is a traditional Chinese herbal medicine commonly used for inflammatory diseases. In ancient, SO is applied for stroke, but mechanisms have not been clear. The purpose is to investigate whether SO exerts neuroprotective effects by reducing microglia-related inflammatory injury after focal cerebral ischemia/reperfusion (I/R).

**Methods:**

SO was extracted using 50% ethanol and quality control was performed by Waters ACQUITY-UPLC CLASS system. The focal cerebral I/R model was established in mice, neurological functions, weight loss and infarct volume was evaluated. Microglial status in penumbra was monitored by immunofluorescence staining and morphology. Mouse primary microglia was subjected to lipopolysaccharide stimulation for triggering inflammatory response, meanwhile microglia-neuron co-culture model was established *in vitro*. Apoptosis and pyroptosis for neurons were demonstrated using TUNEL assay, western blot and ELISA. Inflammatory cytokines were detected by qPCR and ELISA.

**Results:**

SO treatment reduced infarct volume, improved neurological functions, lessened neuronal apoptosis and pyroptosis shown by increased Bcl-2/Bax ratio, decreased cleaved Caspase-3 and suppressed NLRP3/Caspase-1/GSDMD expression after I/R. Moreover, microglial status from pro-inflammation to anti-inflammation was characterized by morphological change and increasing percentage of CD206/Iba1 positive cells after SO treatment. Additionally, SO decreased TLR4 and nuclear-located p65 expression, suppressed IL-1β, IL-6, IL-18, and TNF-α, but increased IL-10 secretion both *in vivo* and *vitro*.

**Conclusion:**

SO reverses pro-inflammatory status of microglia as evidenced by suppression of TLR4/NF-κB cascade, down-regulation of pro-inflammatory cytokines and up-regulation of anti-inflammatory cytokines, which contributes to its neuroprotective against neuronal pyroptosis and apoptosis after ischemic stroke.

## 1 INTRODUCTION

Presently, stroke is the second leading cause of mortality and disability globally, with a dominant incidence in ischemia [1]. Administration in acute ischemic period relies on tissue plasminogen activator to achieve arterial recanalization, but restoring cerebral blood flow may lead to formation of reactive oxygen species that contributes to reperfusion injury. Meanwhile, microglia-responded inflammation is also found to involve in the pathological evolution following stroke [2].

Microglia accounts for 20% of the total glial cell population and distributes throughout the brain in non-overlapping regions, which constantly moves and analyzes the central nervous system (CNS) for immune defense. When stroke occurs, the complex inflammatory response mainly exists in the brain penumbra area following activation of microglia, which presents changes of morphology and gene expression [3]. By analysis on phenotypic and morphological changes, microglia can be determined to be either in pro- or anti-inflammatory status [4, 5]. Toll-like receptor 4 (TLR4) in microglia not only plays key role in inflammatory response resulted by cerebral ischemia, but also appears to be involved in changes of microglial morphology [6, 7]. In the classical nuclear transcription factor kappa B (NF‐κB) pathway of microglia, endogenous and exogenous agonists of TLR4 advances the phosphorylation of IκBα, which is prone to nuclear translocation of NF-κB, and then initiates transcription of genes associated with inflammation and the production of inducible nitric oxide synthase (iNOS) [8]. The pro-inflammatory microglia is characterized by iNOS and NF-κB activation, produces nucleotide binding domain like receptor 3 (NLRP3) inflammasome and releases nitric oxide (NO) and pro-inflammatory cytokines such as interleukin-1β (IL-1β), IL-6, tumor necrosis factor-alpha (TNF-α) [9, 10] . NLRP3 inflammasome can activate caspase-1, which promotes the maturation and secretion of IL-1β [11, 12]. While the anti-inflammatory microglia, marked by molecules production like CD206 or IL-10, supports to tissue repairing and exerts neuroprotective effects in stroke [5]. In lipopolysaccharide (LPS)-stimulated microglia, overexpression of IL-10 reduces NLRP3 inflammasome activity, and inhibits caspase-1 related IL-1β maturation [13]. Therefore, new therapies strategy should be aimed at microglia-mediated anti-inflammatory efficacy for restoring brain function post stroke [14].

Neuronal apoptosis and pyroptosis are two common types of regulated cell death after ischemic stroke, which usually happens in region of ischemic penumbra [15]. As noninflammatory cell death, Bcl-2 and Bax play crucial roles in the regulation of neuronal apoptosis, and caspase-3 participates in this process [16]. Bax, as a pro-apoptotic gene, enhances cell death signals or directly affects apoptosis by activating caspase-3. However, this effect can be reversed by the anti-apoptotic gene Bcl-2. Recent studies have shown that if apoptotic cells are not cleared in time, they will induce further tissue necrosis and inflammation [17, 18]. Pyroptosis is a non-apoptotic inflammatory cell death, which occurs downstream of NLRP3 inflammasome activation. Pyroptosis is mediated directly by gasdermin D (GSDMD), which can be cleaved by pro-inflammatory caspase-1 to forming pyroptotic membrane pores [15]. In addition, activated caspase-1 also cleaves pro-IL-1β and pro-IL-18 into mature IL-1β and IL-18, then release from the GSDMD pores, leading pro-inflammatory responses [19]. Thereby, targeting the two types of regulated cell death therapeutically shows promise in stroke neurological injury.

*Siegesbeckia orientalis L.* (SO), a kind of Chinese herbal medicine, can be traced back to the Ming Dynasty in China for treating stroke. However, the therapeutic mechanism is still not discovered. Studies indicate that SO is used to treat rheumatoid arthritis (RA), hyperuricemia and postoperative cognitive dysfunction due to its anti-inflammatory effect [20-23], and the anti-RA effect is related to decrease the expression of IL-1β and IL-6 in joint muscles [24], inhibit TLR4/NF-κB signaling both *in vivo* and *in vitro* [20, 21]. An interesting study found that SO can control the inflammatory response of BV2 cells and mouse hippocampus through NF-κB pathway to ameliorating perioperative neurocognitive disorders [25]. In this study, we intend to validate the hypothesis whether SO can reverse pro-inflammatory status of microglia to perform neuroprotective action after focal cerebral I/R.

## 2 MATERIALS AND METHODS

### 2.1 Quality Control of SO Extract

16-O-acetyldarutoside and 3,7-dimetoxi-5,3',4'-tri-hidro-xifavonawere purified from the herbal extract using a pre-parative HPLC approach, the purities (>98%) were determined by HPLC analysis and the structures were identified by Nuclear Magnetic Resonance (NMR). Kirenol and darutoside (purities > 98% by HPLC analysis) were purchased from Chengdu Pufei De Biotech Co., Ltd. (Chengdu, China). Acetonitrile was purchased form Merck Company (Darmstadt, Germany) of HPLC grade. All other reagents and chemicals were of analytical grade. Milli-Q water was prepared using a Milli-Q system (Millipore, MA, USA). The herbal material of SO was collected from Ganzhou (Jiangxi province, China) and authenticated by Prof. Yu Hua. The voucher specimens (No. SO-003) were deposited at the Institute of Chinese Medical Sciences, University of Macau, Macao, China.

The powdered SO herb (100 g) was extracted thrice with 50% ethanol (1:10, w/v) for 1 h each under reflux. The combined extracts were filtered with filter paper after cooling and then concentrated under reduced pressure to a appreciate volume. The powdered SO extract (yield: 16.2%) was obtained by lyophilizing the concentrated sample with a Virtis Freeze Dryer (The Virtis Company, New York, USA).

Quantification of 16-O-acetyldarutoside, 3,7-dimetoxi-5,3',4'-tri-hidroxifavona, kerinol and darutoside in SO extract was performed using a Waters ACQUITY-UPLC CLASS system (Waters Corp., Milford, USA) coupled with an ACQUITY UPLC HSS T3 column (150 mm×2.1 mm, 1.8 µm) maintained at 35^o^C. Elution was performed with a mobile phase of A (0.2% phosphoric acid in water) and B (0.2% phosphoric acid in ACN) under a gradient program: 0-5 min, 20% B; 5-15 min, 20-32% B; 15-28 min, 32-55% B. The flow rate was 0.35 mL/min, and the injection volume was 2 μl. The analytes were monitored at the UV wavelength of 215 nm. Between two injections, the column was washed with 100% B for 2 min and equilibrated with the initial mobile phase for 5 min.

### 2.2 Focal Cerebral Ischemia/Reperfusion Model and SO Administration

C57BL/6J (B6) mice (20 to 25 g, male) were selected in this study. The use and care of the mice were in accordance with the guidelines of Animal Advisory Committee of Zhejiang Chinese Medical University (No. 15399) and US National Research Council Guide for the Care and Use of Laboratory Animals (the eighth edition). Mice subjected to transient middle cerebral artery occlusion (tMCAo) in right hemisphere by intraluminal filament technique for 50 mins as previously described [26] was recognized as focal cerebral I/R model. Male C57BL/6 mice were anesthetized with 1% sodium pentobarbital solution. First, make an incision in the middle of the neck, and then carefully expose and dissect the right common carotid artery (CCA), external carotid artery (ECA), and internal carotid artery. Temporarily ligate the right side of the neck and make a small incision in the external carotid artery. Then A nylon monofilament with a silicone tip was inserted from the ECA into the ICA to block the right middle cerebral artery (MCA). After 50 mins of occlusion, the fibers were removed for reperfusion, and the ECA was ligated to close the wound. The sham group only underwent the same procedure of separated blood vessels. Subsequently, the mice were randomly grouped in five: three doses of SO groups, tMCAo and Sham groups. In three doses of SO groups, animals were respectively administered low dose (0.3 g/kg), middle dose (0.6 g/kg) and high dose (1.2 g/kg) of SO extract dissolved in distilled water through intragastric manner for 7 days. In tMCAo and Sham groups, the animals were only given by gavage with the same volume of distilled water and same times.

### 2.3 Modified Neurological Severity Scores (mNSS) and Weight Loss Rate

We used mNSS to evaluate at 1, 3 and 7 day after tMCAo. Neurological function was graded on a scale of 0 to 20 (normal score, 0; maximal deficit score, 20). mNSS consists of motor, sensory, reflex, and balance tests. Higher the score, the more serious damage mice have [27].

In order to observe the weight change of mice after I/R and SO administration, the rate of weight loss was calculated. Weight loss rate (%) = (preoperative weight - postoperative weight) / preoperative weight⨯100%.

### 2.4 Rotor-Rod Test

**Fig. (1):**
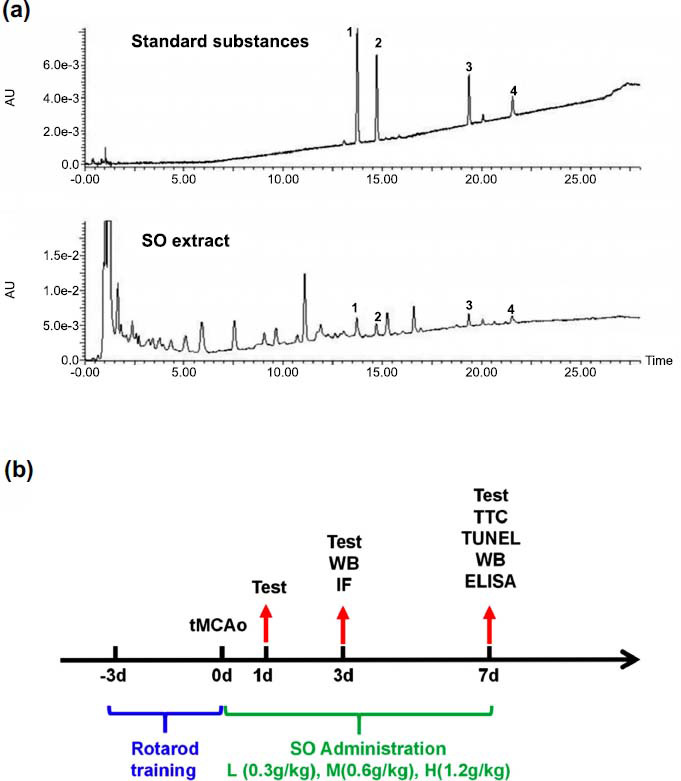
Quality control of SO and animal experiment timeline. (**a**) Comparison of four standards can be detected in SO extract *via* UPLC: 1. 16-O-acetyldarutoside, 2. 3,7-dimetoxi-5,3',4'-tri-hidroxifavona, 3. Kerinol, 4. darutoside. (**b**) Timeline for animal experiments.

Rotor-Rod is a test used to assess sensorimotor coordination and motor learning in rodent models of CNS disorders. Briefly, the mice were placed on a rotating rod with a steady acceleration till to constantly rotate, and the latency to fall was recorded [28]. Three days' training was needed to ensure that all mice had learned the task to the same degree (Fig. **1b**). Mice received three measurements per test, and the average value of latency to fall was calculated.

### 2.5 Grip Strength Test

The Grip Strength test is used to evaluate forelimb motor function and deficit. The mice’s forepaws were placed on a metal bar, which caused the mice would naturally grasp the bar while its tail was gently pulled backwards by an inspector [29]. The maximum strength of the grip prior to grip release was recorded, and the strength value before MCAo was recognized as the baseline. The test was performed with each session consisted of five measurements, and the mice’s average value for each session was presented. The ratio of average value at day 1, 3, 7 after MCAo to the baseline was used to assess the amelioration of grip strength.

### 2.6 Infarction Volume

2,3,5-triphenyltetrazolium chloride (TTC, Sigma-Aldrich) staining was applied for detection of infarction volume post-stroke. The brains collected from Sham and 7d post-I/R mice in each group (n=3) were cut into five slices (2mm per slice), and incubated with 2% TTC PBS buffer at 37°C for 15 mins. Cerebral tissue in the infarcted area presented milky white color, while non-infarcted areas were red. Images were obtained using a digital camera and the infarct volume was analyzed by Image-Pro Plus 6.0 software (Meida Cybernetics). The value of the infarct volume was presented as a percentage of the whole brain. The percentage of infarct volume = total infarct volume / total brain volume × 100%.

### 2.7 Immunofluorescence Staining

Mice of each group were sacrificed by excessive anesthesia, and transcardially perfused with PBS buffer and 4% paraformaldehyde PBS buffer. The brains were cut into 20 μm coronal frozen sections after being gradient dehydrated in 15% and 25% sucrose PBS buffer. The brain sections were incubated with rabbit anti-CD206 (1:500, Abcam), goat anti-Iba1 (1:500, Abcam) and rabbit anti-NeuN (1:500, Abcam) antibodies overnight at 4°C. After being washed with PBS, the sections were incubated with the appropriate secondary antibodies for 2 h in the dark at room temperature. DAPI (1:1000, Bioworld) was used to stain the nucleus. The slides were imaged using Olympus FV3000 microscope, and the images were quantified with Image-Pro Plus 6.0 software.

### 2.8 Cells Co-culture and Dose Determination of SO Extract *in vitro*

Primary microglia and cortical neurons were respectively isolated from the brain of 0~1 day-old C57BL/6J mice and day 15 to 17 embryos of C57BL/6J mice as previously described [30]. Primary cortical neurons were seeded in 24-well plates containing neurobasal medium, and primary microglia was maintained in DMEM supplemented with 10% fetal bovine serum. In order to validate the neuroprotective effect of SO *via* regulation of microglia inflammatory status, neuron-microglia co-culture was needed to be established. To observe whether microglia's DMEM medium can be replaced with neurobasal (neuron medium), we used CCK-8 assay (Dojindo) to compare the vitality of microglia with different culture mediums. Briefly, CCK-8 solution was respectively added to DMEM and neuron medium solution (CCK-8: medium = 1:10) in each culture well for incubation at 37°C for 1h. The absorbance at 450 nm was measured with a microplate reader (TECAN). As shown in Fig. (**S1**) a, after 48 hours, the results of CCK-8 showed that DMEM and neurobasal mediums had no significant influence on microglial survival ability (*p >* 0.05). Therefore, in present study, primary microglia (microglia: neurons = 1:2, as [31] mentioned) were seeded on 0.4 μm pore-sized Transwell inserts (Costar) and neurons were cultured in the low chamber, and neurobasal medium was chosen for co-culture for 24 h.

To explore the optimal dose scope of SO extract for regulating inflammatory status of microglia *in vitro*, we used CCK-8 assay to test normal and LPS-induced microglia vitalities after 24 hours administration of different doses' SO extract. As shown in Figure, it suggested that the doses of 10 μg/ml, 20 μg/ml and 50 μg/ml slightly increased normal cell vitalities. Before 1h of microglia incubation with LPS (100 ng/ml), different doses' SO extract were added into microglia. After 24 hours, it showed the cell vitality increased due to inflammatory stimulation, and the doses of 1μg/ml and 2 μg/ml obviously inhibited cell vitality, while the doses of 10 μg/ml, 20 μg/ml and 50 μg/ml gradually reversed the inhibited trend. Based on the analysis of different doses of SO extract on normal and LPS-induced microglial vitalities, we finally determined 10 μg/ml, 20 μg/ml and 50 μg/ml of SO extract as low, middle and high doses *in vitro*. In present study, the determined three doses' SO extract were added into the upper chamber of transwell with co-culture of microglia and neurons, and after 1 hour, LPS (100 ng/ml) also added into transwell inserts, which was identified as low, middle and high doses groups. Simultaneously, co-cultured cells without LPS simulation was set as control group and co-cultured cells which only 50 μg/ml SO extract was added into were recognized as drug control. After 24 hours, related experimental indexes in five groups were detected.

### 2.9 TUNEL Assay

*In vivo*, TUNEL (Beyotime) was co-stained with NeuN to label apoptotic neurons. After brain slices incubated with NeuN and corresponding fluorescent secondary antibody, 50 μl of TUNEL assay solution was added and incubated at 37°C for 60 mins in the dark. Washed with PBS three times. *In vitro*, neurons of each co-cultured system were also stained with TUNEL, and then labeled with DAPI as nucleus. The brain sections and cell wells were imaged using Leica, DM6B microscope, and the images were quantified with Image-Pro Plus 6.0 software.

### 2.10 Neurons Viability and Nitric Oxide Detection

In order to detect whether the protective efficacy of SO extract on neurons in LPS-induced condition is related to regulation of microglia inflammatory status, CCK-8 was used to assess cell survival. The experimental steps were performed similarly as mentioned above.

Levels of NO derivative nitrite in five groups were determined with the Griess reaction. A nitrite detection kit (Beyotime) was used according to instructions provided by the manufacturer. The samples from upper chamber medium were assayed in triplicate, and a standard curve using NaNO_2_ was generated for each experiment for quantification. Briefly, 50 μl of medium or standard NaNO_2_ was mixed with 100 μl of Griess Reagent I and II in a 96-well plate. After 15 min, optical density was measured in a microplate reader (TECAN) at 540 nm. NO production is expressed as a percentage of LPS treatment alone. The data are representative results obtained from three independent experiments.

### 2.11 Real-Time PCR

Primary microglial cells (3×10^4^ pcs / ml) were administered with different doses of SO extract for 1 h, and subsequently stimulated with 100 ng/mL LPS for 6 h. The total RNA was extracted using Trizol reagents (Invitrogen) and then reverse-transcribed into cDNA with the Prime Script RT regent kit (Takara) according to protocol. Real-time PCR was performed on a Step One Plus PCR system (Applied Biosystems, USA) using a SYBR Green Kit (Applied Biosystems). The primers and sequences derived from the relative mRNA expression were analyzed using the formula 2^-△△Ct^. The primer sets used as follow: IL-1β F: 5’-AAGCCTCGTGCTGTCGGACC-3’, R: 5’-TGAGGCC-CAAGGCCACAGGT-3’; IL-6 F: 5’-GCTGGTGACAACC-ACGGCCT-3’, R: 5’-AGCCTCCGACTTGTGAAGTGGT-3’; TNF-α F: 5’-CAAGGGACAAGGCTGCCCCG-3’, R: 5’-GCAGGGGCTCTTGACGGCAG-3’; iNOS F: 5’-CAG-CTGGGCTGTACAAACCTT-3’, R: 5’-CATTGGAAGTG-AAGCGTTTCG-3’; IL-10 F: 5’-GCCAGAGCCACATGC-TCCTA-3’, R: 5’-GATAAGGCTTGGCAACCCAAGTAA-3’; GAPDH F: 5’-GCCAAGGCTGTGGGCAAGGT-3’, R: 5’-TCTCCAGGCGGCACGTCAGA-3’.

### 2.12 ELISA

The levels of inflammatory cytokines (IL-1β, IL-6, IL-18, TNF-α) in cell and tissue supernatants were determined by ELISAs (CUSABIO), and IL-10 was detected by ELISA kit from FcMACS. The experimental steps were strictly performed according to the different manufacturers’ manual.

### 2.13 Western Blot

Cytoplasmic and nuclear proteins from primary microglia and brain tissue were extracted using NE-PER nuclear and cytoplasmic extraction reagents (Thermo) according to the manufacturer’s instructions. The total protein was quantified by a BCA protein assay kit (Thermo). The proteins in each sample were separated with 10 or 12% sodium dodecyl sulfate polyacrylamide (SDS-PAGE), and transferred onto a PVDF membrane (Millipore). After blocking with 5% BSA in TBST for 1 h at room temperature, the membranes were incubated with the appropriate primary antibodies (TLR4, p65, NLRP3, Bcl-2, Bax 1:1000, from CST; caspase-1 1:1000 from abclonal; cleaved caspase-3 1:1000, β-actin, Lamin B1 1:5000, from Bioworld; GSDMD 1:5000, from Abcam) overnight at 4°C. Thereafter, the protein bands were incubated with secondary antibody at room temperature for 1 h. The proteins were detected with an enhanced chemiluminescence detection system (ECL), and the images were scanned using the Gel-Pro system (Tanon). The intensity of the bands was quantified with Image J software. The analysis was conducted in triplicate for each target.

### 2.14 Statistical Analysis

All results were expressed as means ± standard deviation. Two-way repeated measures analysis of variance (two-way-ANOVA) was performed to determine the interaction effect between group and time period on related mice neurological function and weight loss evaluation, and one-way ANOVA was used to analyze other data followed by tukey test for multiple comparisons. Differences were considered to have statistical significance at *p <* 0.05.

## 3 RESULTS

### 3.1 Quality Control Results of SO Extract

The representative compounds of 16-O-acetyldarutoside, 3,7-dimetoxi-5,3',4'-tri-hidroxifavona, kerinol and darutoside in SO extract were quantitatively determined using the UPLC-PDA methods. As illustrated in Fig. (**1**) a, all detected compounds could be chromatographically separated without interferences. Moreover, the contents of 16-O-acetyldarutoside, 3,7-dimetoxi-5,3',4'-tri-hidroxifavona, kerinol and darutoside in SO extract were quantified to be 0.695 ± 0.016%, 0.218 ± 0.009%, 0.530 ± 0.027% and 0.483 ± 0.017%, respectively.

### 3.2 SO Treatment Improved Neurological Function and Reduced Body Weight Loss

**Fig. (2):**
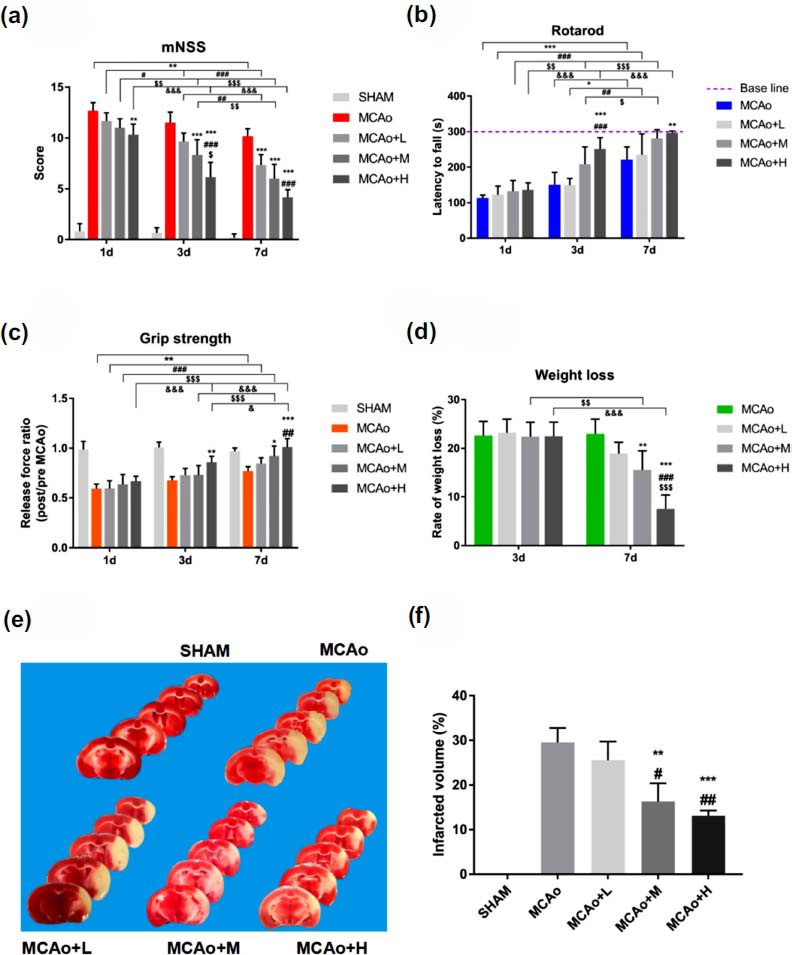
SO treatment ameliorates neurological function, lessens weight loss and cerebral infarct volume in mice with I/R. (**a**) mNSS, (**b**) rotarod, (**c**) grip strength and (**d**) weight loss were assessed in mice. (**e**) Representative TTC stained coronal sections of mice brain in different groups at 7d after I/R. (**f**) Quantitative analysis of infarct volume is performed. Data are means ± SD (n=8, per group in a-d; n=3, per group in e). *p < 0.05, **p < 0.01, ***p < 0.001 *vs.* MCAo; ^#^*p <* 0.05, ^##^*p <* 0.01, ^###^*p <* 0.001 *vs.* MCAo+L; ^$^*p <* 0.05, ^$$^*p <* 0.01, ^$$$^*p <* 0.001 *vs.* MCAo+M; ^&^*p <* 0.05, ^&&&^*p <* 0.001 *vs.* MCAo+H.

A total of 82 mice were used in this study, and 7 of them died, including 3 mice died unexpectedly during the MCAo surgery and 4 mice died after MCAo surgery (2 in MCAo group, 1 in MCAo+L group, and 1 in MCAo+M group). The overall mortality rate was around 8.5% in mice study. The neurological function of the mice after tMCAo showed obvious defects, which was manifested by an increase in the mNSS score (Fig. **2a**). There was only one difference on improvement of mNSS score between high dose group and MCAo group after 1 day of stroke (*p <* 0.01). At day 3 and 7, the degree of amelioration in the SO administration groups increased significantly, especially in middle-high dose group (*p <* 0.001 *vs.* MCAo and low dose). With the days lasting, the scores of each group gradually decreased, notably in the high dose group. It suggested that SO treatment could repair the neurological deficit in stroke mice.

As shown in Fig. (**2b**), mice of each group were trained to stably walk on the rotating rod for 300 seconds before surgery. After tMCAo, the time mice spent on the rods was significantly shortened. Then began to gradually extend, obviously in high dose group (*p <* 0.001 *vs.* MCAo and low dose at day3; *p <* 0.01 *vs.* MCAo at day7). In terms of grip strength, high dose of SO group at day 3 (*p <* 0.01 *vs.* MCAo), middle dose (*p <* 0.05 *vs.* MCAo) and high dose (*p <* 0.001 *vs.* MCAo, *p <* 0.01 *vs.* low dose) groups at day 7, showed obviously ameliorated effects on grip strength (Fig. **2c**).

In addition, we also found that SO treatment could attenuate the weight loss of mice after stroke, which was also used as an indicator of rehabilitation (Fig. **2d**). The degree of body weight loss had no distinct difference between MCAo and SO administration groups at day 3 post-surgery. However, at day 7, the weight loss in middle-high dose group decreased significantly (*p <* 0.01 middle dose *vs.* MCAo; *p <* 0.001 high dose *vs.* other groups), and there was also a significant difference in middle-high dose group at day 7 compared with at day 3 (*p <* 0.01).

### 3.3 SO Treatment Reduced Cerebral Infarction Volume, Attenuated Neuronal Apoptosis and Pyroptosis

From the 7 day's brain tissue TTC staining section, the amelioration of infarction volume in low dose group was not obvious compared with tMCAo group. However, middle-high dose group notably lessened the infarcted area (Fig. **2e**). Quantitative analysis suggested the degree of improvement in middle-high dose group were higher than that in low dose group (*p* < 0.05), and the efficacy in high dose group was the most significant (Fig. **2f**).

**Fig. (3):**
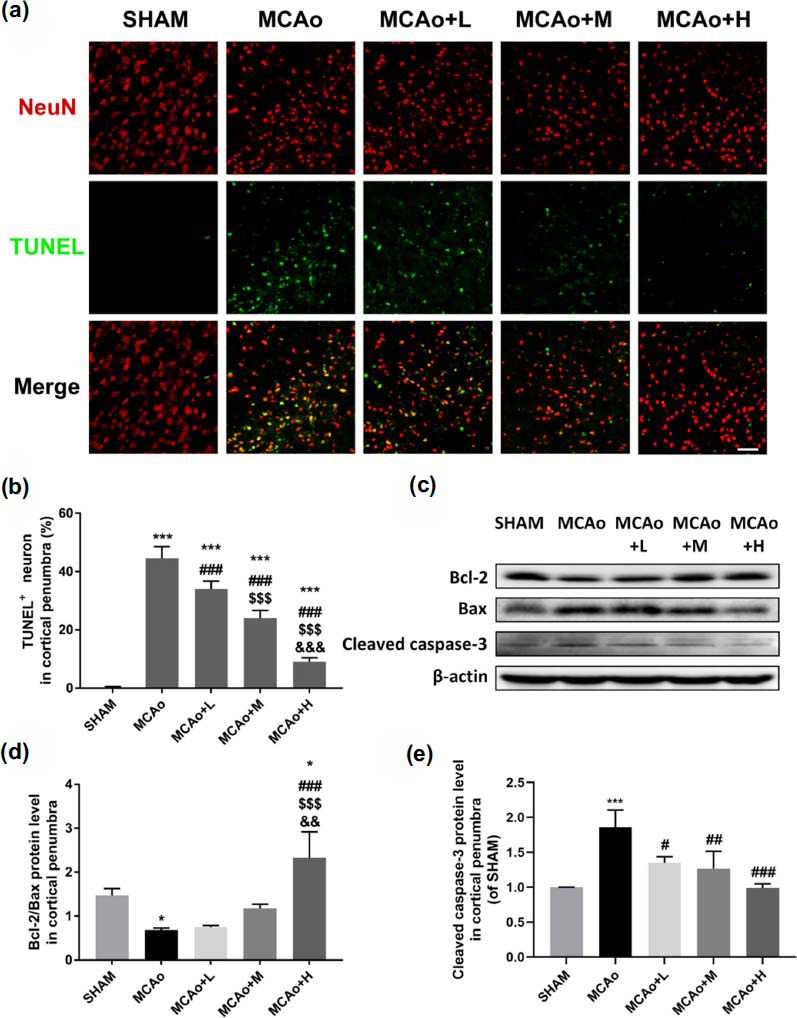
SO treatment alleviates neuronal apoptosis in ischemic penumbra. (**a**) NeuN (red) and TUNEL (green) are co-stained (yellow) in the penumbra of the ischemic cortex at day 7 I/R mice. (**b**) Statistics on the proportion of TUNEL^+^ neuron expression marked as apoptotic cells. (**c**) Representative western blot bands of Bcl-2, Bax, cleaved caspase-3 in penumbra brain tissue, and quantitative statistics of Bcl-2/Bax (**d**), cleaved caspase-3 (**e**) expressions. Data are means ± SD (n = 3 per group). *p < 0.05, ***p < 0.001 *vs.* SHAM; ^#^*p <* 0.05, ^##^*p <* 0.01, ^###^*p <* 0.001 *vs.* MCAo; ^$$$^*p <* 0.001 *vs.* MCAo+L; ^&&^*p <* 0.01, ^&&&^*p <* 0.001 *vs.* MCAo+M. Scale bar = 50 μm.

In order to observe the level of damaged neurons, we stained the penumbra cortex tissue with TUNEL (labeling apoptosis, green) and NeuN (neuronal marker, red) co-immunofluorescence. After 7 days of cerebral I/R in mice, except for the sham group, neurons in ischemic penumbra cortex of each group showed different degrees' apoptosis, manifested with shrinking nuclei (Fig. **3a**). Compared with MCAo group, SO administration notably reduced the proportion of TEUNEL & NeuN^+^ cells, and the improved efficacies were in a dose-dependent manner (Fig. **3b**, *p <* 0.001, high dose *vs.* middle-low dose). We also detected the cascade of apoptosis-related Bcl-2/Bax/Caspase3 in ischemic penumbra (Fig. **3c**). Western blot results showed the ratio of Bcl-2/Bax in MCAo group decreased significantly (*p <* 0.05 *vs.* Sham, Fig. **3d**) and cleaved caspase-3 in MCAo group was obviously increased (*p <* 0.001 *vs.* Sham, Fig. **3e**). Caspase-3 decreased and the ratio of Bcl-2/Bax increased in a dose-dependent manner after SO administration. It suggested SO treatment can attenuate neuronal apoptosis *via* regulation of Bcl-2/Bax/Caspase3 pathway.

**Fig. (4):**
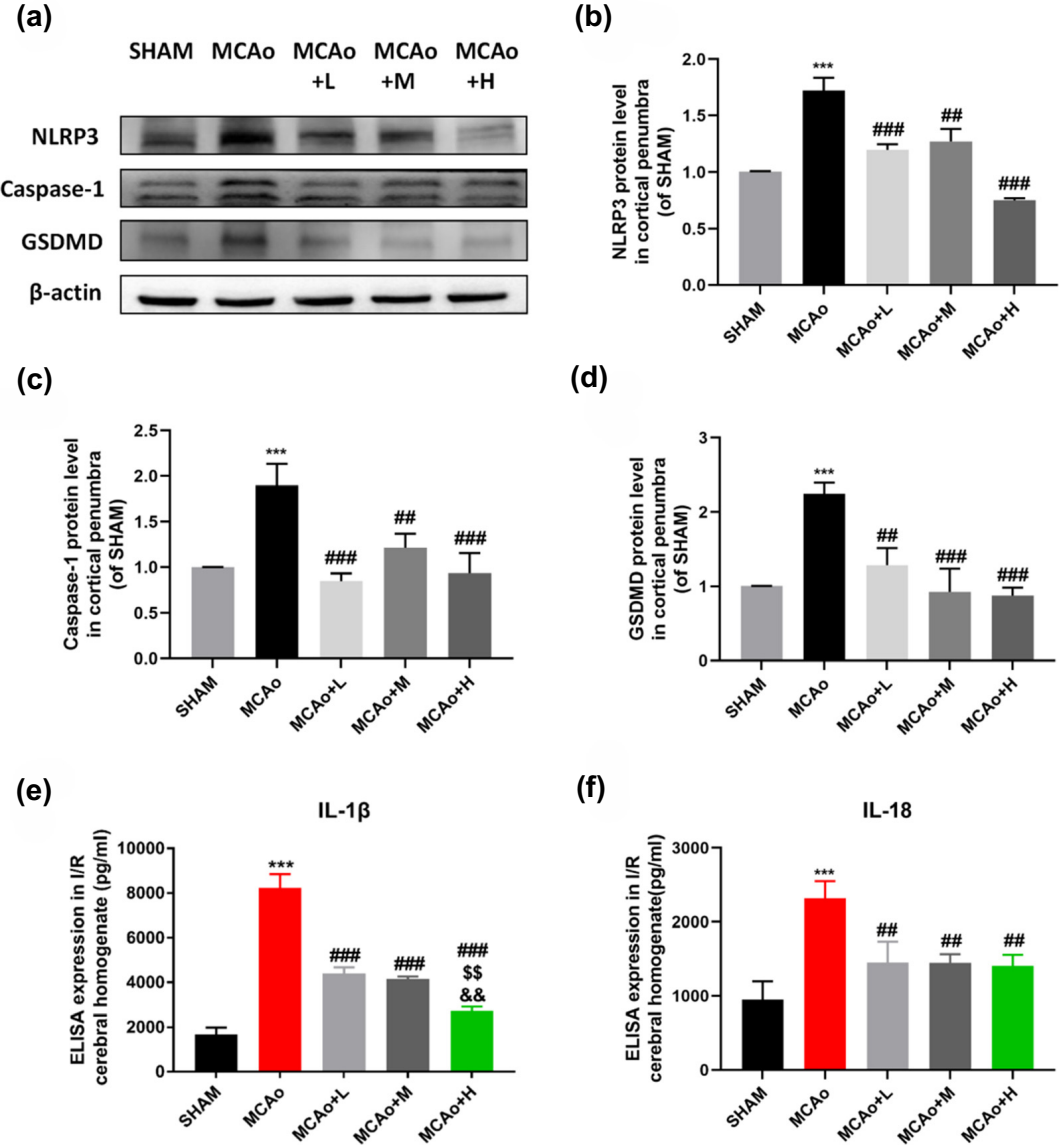
SO treatment reduces NLRP3 inflammasomes and inhibits neuronal pyroptosis in ischemic penumbra. (**a**) Representative western blot bands: NLRP3, Caspase-1, GSDMD expression in penumbra brain tissue at day 3 I/R mice. Gray value quantitative statistics of NLRP3 (**b**), Caspase-1 (**c**), GSDMD (**d**). And cerebral infarction tissue homogenate detection by ELISA: IL-1β (**e**), IL-18 (**f**). Data are means ± SD (n = 3 per group). ***p < 0.001 *vs.* SHAM; ^##^*p <* 0.01, ^###^*p <* 0.001 *vs.* MCAo; ^$$^*p <* 0.01 *vs.* MCAo+L; ^&&^*p <* 0.01 *vs.* MCAo+M.

Pyroptosis is another common regulated cell death associated with inflammation in stroke. As expected, western blot results displayed that NLRP3/Caspase-1/GSDMD pyroptosis cascade was significantly activated in MCAo group (Figs. **4a**-**d**, *p <* 0.001), accompanying increased release of IL-1β and IL-18 in ischemic brain tissue by ELISA detection (Figs. **4e** and **f**, *p <* 0.001). After SO intervention, GSDMD was inhibited, IL-1β and IL-18 were reduced, especially pronounced in middle and high dose groups.

### 3.4 SO Treatment Reversed Pro-inflammatory Status of Microglia

**Fig. (5):**
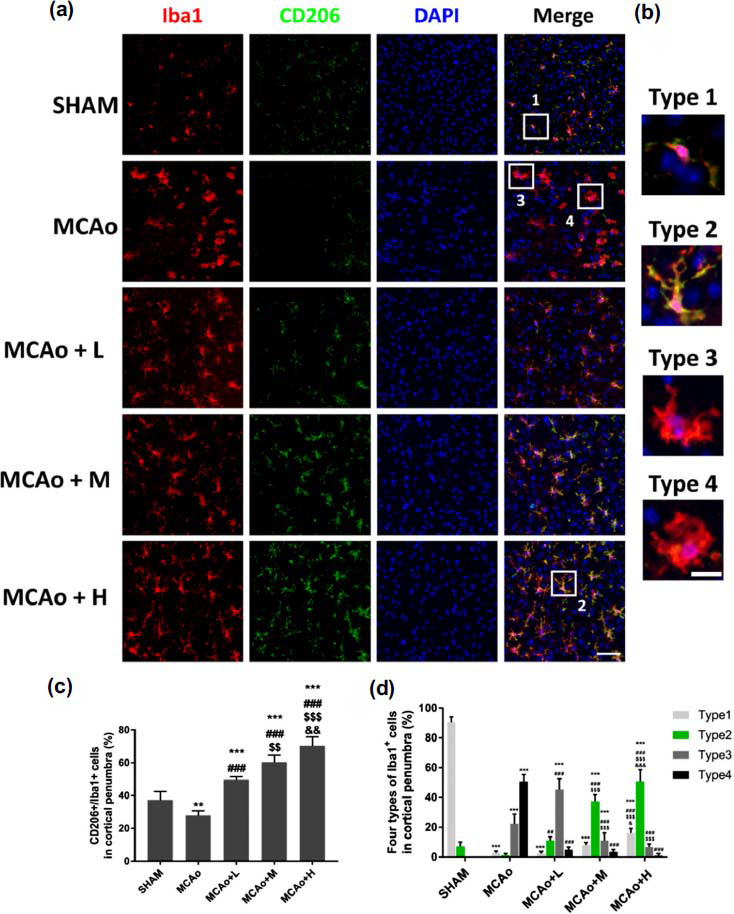
SO treatment regulates inflammatory status of microglia in infarcted cortex penumbra at day 3 I/R mice. (**a**) Immunofluorescence staining of Iba1 (red) and CD206 (green) in the peripheral zone of cerebral cortex infarction in each group, DAPI (blue) represents the nucleus, bar =50 μm. (**b**) Typical four phenotypes of Iba1^+^ microglia (red) merged with CD206 (yellow) are enlarged, bar =25 μm. (**c**) Proportion of CD206 positive microglia in each group. (**d**) Statistics on the proportion of 4 phenotypes of microglia in each group. Data are means ± SD (n = 3 per group). **p < 0.01, ***p < 0.001 *vs.* SHAM; ^##^*p <* 0.01, ^###^*p <* 0.001 *vs.* MCAo; ^$$^*p <* 0.01, ^$$$^*p <* 0.001 *vs.* MCAo+L; ^&^*p <* 0.05, ^&&^*p <* 0.01, ^&&&^*p <* 0.001 *vs.* MCAo+M.

Iba1 is a biomarker expressed by microglia. CD206 is a marker of anti-inflammatory microglia (Fig. **5**). After co-staining Iba1 with CD206, it was found that different morphologies of microglia expressed or not expressed CD206 around the ischemic penumbra (Figs. **5a** and **b**). According to [7], we divided microglia into four types: type 1 with less dendritic branches, which might be the resting microglia, mainly expressed in sham group; Type 2 microglia were activated, and mainly characterized by an increase in dendritic branches. We observed this type could be co-stained with CD206 to play an anti-inflammatory effect in local. The type 2 microglia could be seen in the sham group, but barely saw in MCAo group. With enhancing the dose of SO, the proportion of such type cells in penumbra gradually increased, especially in middle-high dose group (Figs. **5b** and **d**; *p <* 0.001); Type 3 microglia’s body began to become larger, the dendrites became thicker and shorter, and gradually transitioned to type 4: a round cell body with almost no dendrite (Figs. **5a** and **b**). Three days after I/R, type 3 and 4 of microglia were abundantly expressed in the ischemic cortex and could not be confocal with CD206. We concluded these two types may aggravate the local inflammatory response. To confirm this, we counted the proportion of CD206 positive microglia. With the increase of SO dose, the ratio of CD206 positive anti-inflammatory microglia also heightened (Fig. **5c**, *p <* 0.01). Additionally, after administration of SO on mice, the proportion of type 4 microglia decreased (Figs. **5a** and **d**, *p <* 0.001 *vs.* MCAo).

### 3.5 SO Treatment Regulated the Levels of Inflammatory Cytokines, Suppressed TLR4/NF-κB/NLRP3 Pathway

**Fig. (6):**
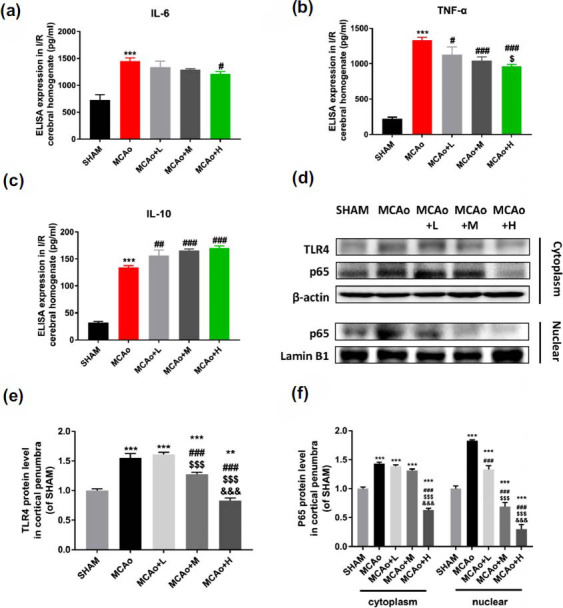
SO administration against post I/R inflammatory injury through TLR4/NF-κB pathway. ELISA detection of cerebral infarction tissue homogenate: IL-6 (**a**), TNF-α (**b**) and IL-10 (**c**). (**d**) Representative western blot bands: TLR4, p65 expression in cytoplasm and p65 in nucleus of penumbra brain tissue. β-actin and Lamin B1 act as cytoplasmic and nuclear internal reference, respectively. Gray value quantitative statistics of TLR4 (**e**), p65 (**f**). Data are means ± SD (n = 3 per group). **p < 0.01, ***p < 0.001 *vs.* SHAM; ^#^*p <* 0.05, ^##^*p <* 0.01, ^###^*p <* 0.001 *vs.* MCAo; ^$^*p <* 0.05, ^$$$^*p <* 0.001 *vs.* MCAo+L; ^&&&^*P <* 0.001 *vs.* MCAo+M.

Firstly, the levels of pro-inflammatory factors: IL-6, TNF-α and anti-inflammatory factor IL-10 in the ischemic penumbra cerebral tissue homogenate were detected by ELISA. The results showed the four pro-inflammatory cytokines greatly increased after cerebral ischemia (Fig. **6a** and **b**, *p <* 0.001 *vs.* sham). Nevertheless, the secretions of IL-6 and TNF-α decreased after SO intervention. Meanwhile, IL-10 gradually rose with dose-dependence of SO (Fig. **6c**, *p <* 0.01 *vs.* MCAo). Additionally, it showed that I/R activated TLR4, promoted p65 nuclear transfer (Figs. **6d**-**f**). However, SO administration reversed the change of TLR4/NF-κB pathway in dose-dependent manner.

**Fig. (7):**
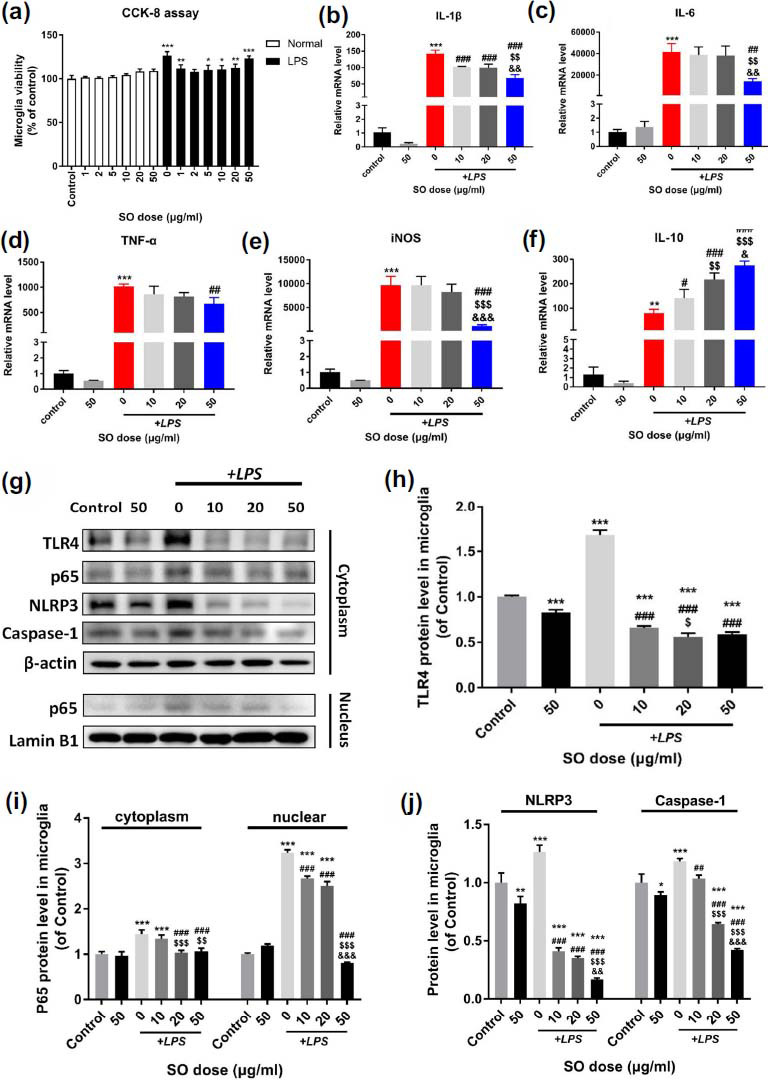
SO treatment reduces pro-inflammatory response and NLRP3 inflammasomes expression through TLR4/NF-κB cascade in LPS-stimulated primary microglia. (**a**) CCK-8 assay on the cell vitality of normal cultured and LPS-stimulated primary microglia by different doses of SO. (**b-f**) SO interfered with the expressions of IL-1β, IL-6, TNF-α, iNOS and IL-10 mRNA in primary microglia stimulated by LPS. (**g**) Representative western blot bands: TLR4, p65, NLRP3, Caspase-1 expression in cytoplasm and p65 in nucleus of primary microglia. β-actin and Lamin B1 act as cytoplasmic and nuclear internal reference, respectively. (**h-j**) Gray value quantitative statistics of TLR4, p65, NLRP3, Caspase-1. Data are means ± SD. *p < 0.05, **p < 0.01, ***p < 0.001 *vs.* Control; ^#^*p <* 0.05, ^##^*p <* 0.01, ^###^*p <* 0.001 *vs.* LPS; ^$^*p <* 0.05, ^$$^*p <* 0.01, ^$$$^*p <* 0.001 *vs.* LPS+10; ^&^*p <*0.05, ^&&^*p <* 0.01, ^&&&^*p <* 0.001 *vs.* LPS+20.

We further validated the regulated inflammatory effects of SO on LPS-induced primary microglia *in vitro*. Firstly, we applied CCK-8 assay to detect viability rate of normal cultured microglia and LPS stimulated microglia after intervention with concentration gradient SO. The results showed that after LPS+SO intervention, there was a certain increase in microglia viability (Fig. **7a**). We selected 10, 20, and 50 μg/ml as intervention doses for SO *in vitro* experiments. From the results analysis of mRNA, the expressions of IL-1β, IL-6, TNF-α, iNOS and IL-10 significantly increased under LPS stimulation (Figs. **7b**-**f**, *p <* 0.01 *vs.* control). While, SO treatment could interfere with the pro-inflammatory effects of LPS by further promoting IL-10 expression in a dose dependent manner (Fig. **7f**). Subsequently, it showed the cascade of TLR4/NF-κB/NLRP3/Caspase-1 in microglia was distinctly activated after LPS incubation, but SO administration evidently attenuated the classic pro-inflammatory pathway compared with control group (Figs. **7g**-**j**).

### 3.6 SO Exerted Neuroprotective effects by Regulating Inflammatory Response and Oxidative Stress of Microglia, thereby Inhibiting Neuronal Apoptosis and Pyroptosis

**Fig. (8):**
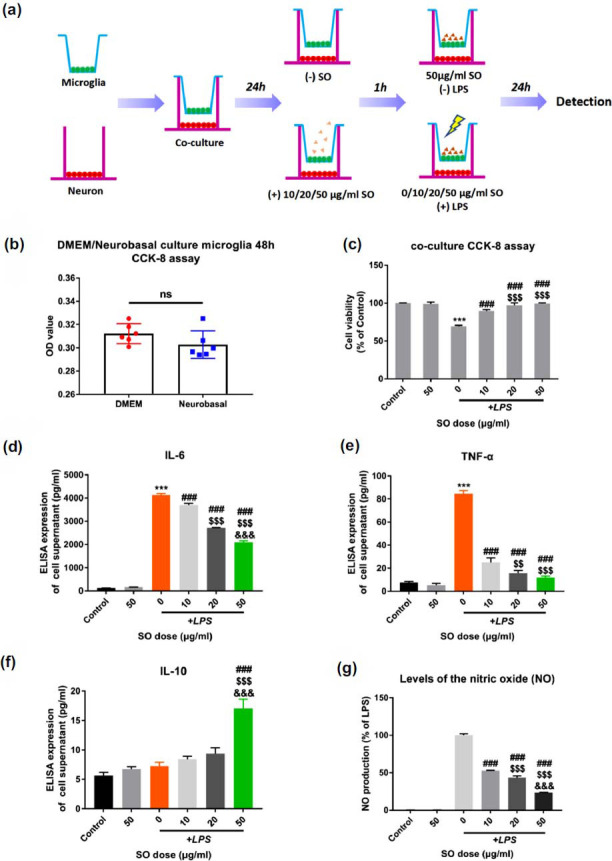
SO treatment attenuates inflammation and oxidative stress levels in LPS-stimulated primary microglia and neurons co-culture system and improves neuronal survival. (**a**) Experimental flow chart of primary microglia and neurons co-culture *in vitro*. (**b**) CCK-8 assay on cell viability of primary microglia after 48 hours cultures in DMEM and Neurobasal. (**c**) Cell viability of neurons below co-cultured system. (**d-f**) The levels of IL-6, TNF-α and IL-10 in co-culture supernatant are detected by ELISA. (**g**) Griess reaction detects the content of nitric oxide in the supernatant of co-culture. Data are means ± SD. ***p < 0.001 *vs.* Control; ^###^*p <* 0.001 *vs.* LPS; ^$$^*p <* 0.01, ^$$$^*p <* 0.001 *vs.* 10+LPS; ^&&&^*p <* 0.001 *vs.* 20+LPS.

In this study, we established microglia-neuron co-culture *in vitro* to validate the neuroprotective effects of SO (Fig. **8a**). To compare if cultured microglia with neuronal medium (neurobasal) and DMEM existed difference of vitality, we tried to culture microglia with neurobasal medium. After 48 hours, the results of CCK-8 showed that there was no significant survival influence on microglia in DMEM and neurobasal groups (Fig. **8b**). Therefore, we determined to use neuronal medium for co-culture experiment. In the subsequent assays of CCK-8 on neurons of lower chamber, SO treatment significantly enhance the survival rate (Fig. **8c**). ELISA detection of the supernatant in the lower chamber also showed that SO treatment suppressed the secretion of pro-inflammatory factors IL-6, TNF-α (*p <*0.01 *vs.* LPS group), and elevated the level of anti-inflammatory factor IL-10 in dose-dependent manner after LPS stimulation (Figs. **8d**-**f**). At the same time, we found that SO can significantly reduce the production of NO by LPS stimulated microglia in a dose-dependent manner (Fig. **8g**, *p <*0.001).

**Fig. (9):**
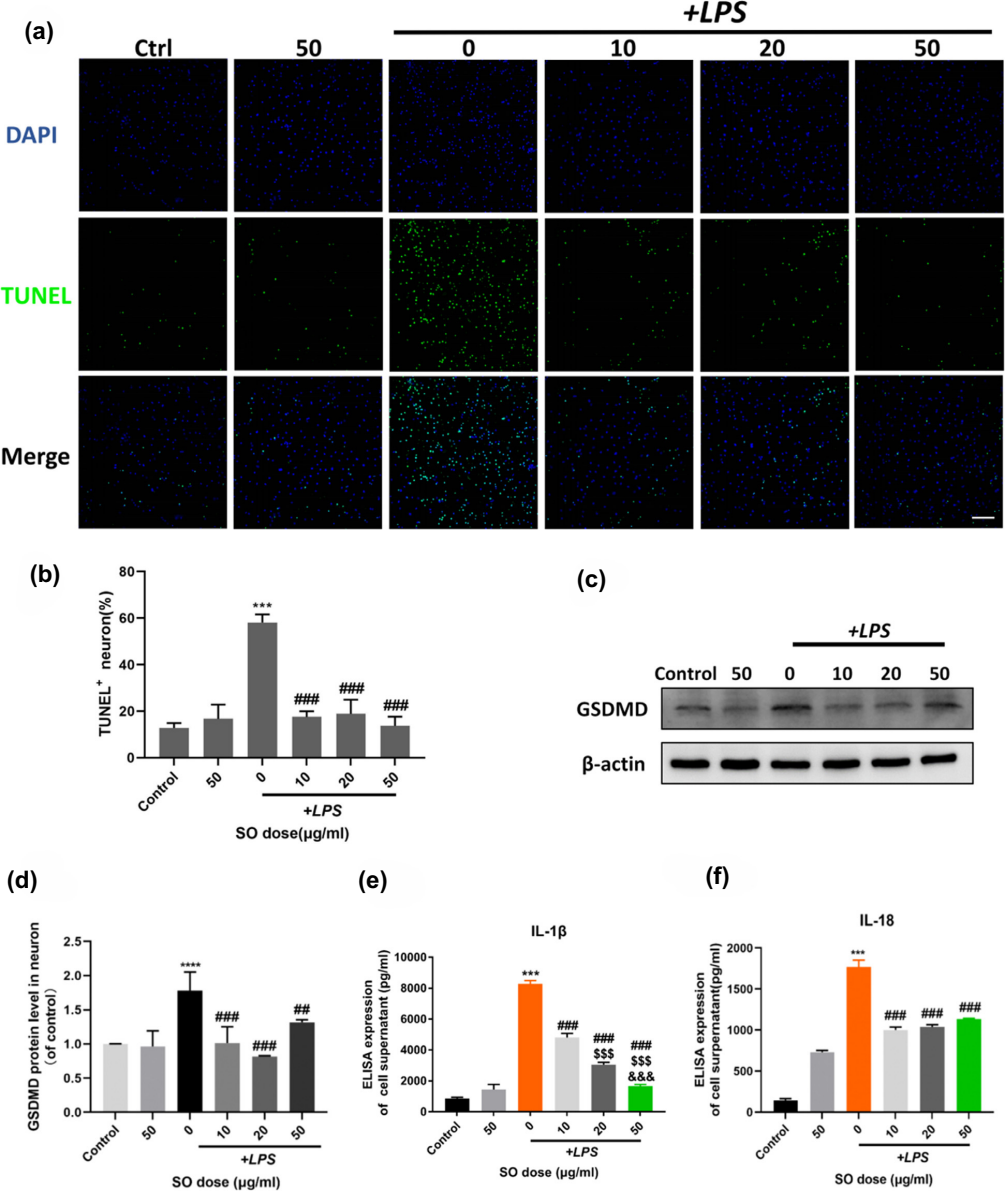
SO treatment mitigates apoptosis and pyroptosis of neurons below the co-culture system. (**a**) Cell nucleus (DAPI, blue) and TUNEL (green) are co-stained in primary neurons below the co-culture system. (**b**) Statistics on the proportion of TUNEL^+^ neuron expression marked as apoptotic cells. (**c**) Representative western blot bands of GSDMD expression in neurons below the co-culture system. (**d**) Gray value quantitative statistics of GSDMD. (**e**, **f**) The levels of IL-1β and IL-18 in supernatant at the lower chamber are detected by ELISA. Data are means ± SD. ***p < 0.001 *vs.* Control; ^##^*p <* 0.01, ^###^*p <* 0.001 *vs.* LPS; ^$$$^*p <* 0.001 *vs.* 10+LPS; ^&&&^*p <* 0.001 *vs.* 20+LPS. Scale bar = 100 μm.

Next, we observed through TUNEL staining that neuron apoptosis of lower chamber in the co-culture system was significantly reduced after SO intervention (Figs. **9a** & **b**, *p <* 0.001 *vs.* LPS group). Meanwhile, it was found that the pyroptosis marker GSDMD with IL-1β and IL-18 secretion were significantly increased in co-cultured neurons after LPS stimulation, however, this trend was reversed after SO intervention (Fig. **9c**-**f**). Interestingly, when SO added in LPS stimulated normal cultured primary neurons without microglia, there is no such protective effect (Fig. **S1**), indicating the microglia-dependent neuroprotective effect of SO.

## 4 DISCUSSION

The present study confirms the neuroprotective effects of SO treatment against cerebral ischemic injury, shown by suppressed neuroinflammation, decreased neuronal apoptosis and pyroptosis, reduced cerebral infarct volume and improved neurological function. The inhibition of microglial TLR4/NF-κB/NLRP3 cascade and the consequent reversion of that pro-inflammatory status of microglia contribute to this neuroprotection effect conferred by SO. These results provide insights into the molecular basis of SO treatment after cerebral I/R.

Inflammation is a devastating pathophysiological process. Microglia is an essential cell type contributing to inflammatory responses in brain, and will be activated under different types of nervous disease. Evidence indicates that microglia can be classified into various subtypes from pro-inflammatory M1 to anti‐inflammatory M2 [3]. The conversion of microglia from M1 to M2 can suppress post-stroke neuronal loss and reduce neurological deficits, and this phenomenon is related to TLR4/NFκB cascade [32]. Additionally, the morphology of microglia also changes under different environmental conditions. Under physiological condition, microglia displays with a small cell body, fine and few branches, which is called as “resting microglia”; When onset of cerebral ischemia, microglia happens to dynamic morphological changes, characterized by retracting and thickening of the branches, and hypertrophy of the cell body [5, 7]. With the alteration of morphology, the microglia are undergoing pro-/anti-inflammatory transformation. In this study, we observed microglia morphological changes post-I/R in ischemic cerebral cortex, and found the cell body became larger and the dendrites became thicker and shorter. However, the morphology was switched by middle-high dose of SO, characterized by an anti‐inflammatory M2 phenotype (Type 2 microglia) with smaller nucleus and longer branches (Iba1 double stained with CD206, Figs. **5a**, **b** and **d**), suggesting the regulatory efficacy of SO on microglial inflammatory status after stroke.

The crucial role of TLR4 in the pathogenesis of stroke has been demonstrated, especially among microglia [3, 33]. Cerebral ischemia can up-regulate TLR4 expression, leading to translocation of NF‐κB to the nucleus, which participates in the transcription of inflammatory factors [34]. However, the results can be inhibited by TLR4 antagonist, which decreases the volume of cerebral infarction, protects BBB integrity, and improves neurological function in I/R mice [7, 35]. Literature evidence that the lessened effect of pro-inflammatory cytokines in ischemic brain tissue and LPS-stimulated microglia are related to inhibition of TLR4 [7, 36]. Here, we confirm SO treatment can suppress the activation of TLR4, thereby contributes to inhibiting p65 nuclear transfer and NLRs inflammasome formation both *in vivo* and vitro.

NLRP3 inflammasome is one of the most representative NLRs inflammasome, mainly expressed in microglia [37]. NLRP3 inflammasome can activate caspase-1 state to cleaved, which in turn exposes the N-terminal of GSDMD for pore formation in cell membrane and promotes secretions of IL-1β and IL-18, causing serious of outcomes in stroke [38, 39]. Researches indicate that suppression of TLR4/NF-κB signaling reduces expression of NLRP3 and GSDMD, which is further effectively improved functional recovery caused by pyroptosis [4, 40]. Inhibition of caspase-1 can attenuate NF-κB activity, not only prevents glial related pyroptosis [4], but also reduces the expression of pro-inflammatory factors, advances transformation of microglia to the anti-inflammatory type, thereby contributes to attenuation of inflammatory injury after stroke [41]. Our results demonstrate that SO administration significantly decreased the expressions of NLRP3 and caspase-1 *in vivo* and *vitro*. In addition, SO also reduced the expression of GSDMD in cerebral ischemic penumbra. Furthermore, by intervening LPS stimulated microglia and neurons co-culturing *in vitro*, the expression of GSDMD in neurons was also inhibited by SO. Interestingly, we found that the expression of GSDMD in the high dose group of SO both *in vivo* and *in vitro* slightly increased compared to the middle dose group, although not statistically significant. We speculate that high dose SO may influence GSDMD through other pathways. This also sparks our interest in future research. However, this does not affect proving SO exerts neuroprotective effects by inhibiting post stroke inflammatory storms, thereby constricts neuronal pyroptosis.

After onset of ischemic stroke, the inflammatory reaction also aggregates neuronal apoptosis in penumbra. On the contrary, if apoptotic cells cannot be cleared in time by microglia, it will induce new inflammatory responses, and activated microglia by inflammation can phagocytize stressed but “alive” neurons resulted in further delayed neuronal loss [18]. Therefore, attenuation of pro-inflammatory status of microglia can reduce infarct volume and ameliorate neurological function. Our study exactly demonstrates SO has the advantage of reversing pro-inflammatory status of microglia. Study has reported that two classical apoptosis-regulating genes involved in apoptosis. Bcl-2 overexpression can inhibit the release of mitochondrial cytochrome C that induces apoptosis, and Bax has the opposite effect. Cell apoptosis is accelerated when Bcl-2/Bax ratio decreases, and *vice versa* [15]. Meanwhile, Bax can be considered as a cell death trigger for ischemic neurons by eliciting caspase-3 activation *via* the mitochondrial pathway [16]. The expression of caspase-3 is up-regulated both in animals and human brain after stroke [42]. Furthermore, when gene knockout and pharmacological inhibition of caspase-3, neuroprotective effects are manifested both *in vitro* and *in vivo* [15]. Our results show that SO treatment can significantly reduce the neuronal apoptosis in cortical penumbra, and the mechanisms may relate to increase of Bcl-2/Bax ratio and caspase-3 inactivation in mice with cerebral I/R.

In stroke, activated microglia cause neuronal damage *via* producing free radicals and various disadvantageous cytokines [3]. Especially, the pro-inflammatory phenotype of microglia induced by ischemia and LPS is characterized by increasing productions of iNOS, IL-1β, IL-6, TNF-α, reactive oxygen species (ROS) and NO resulted in the dysfunction of the CNS [43, 44]. Nevertheless, evidence indicates that microglia also has beneficial aspect for the neuroprotection after cerebral ischemia. The anti-inflammatory phenotype of microglia promotes tissue rehabilitated properties *via* increased production of IL-10 [5]. As a gas signaling molecule, NO participates in regulation of cardiovascular, immune, and nervous systems. Physiological levels of NO have neuroprotective effects. Once overproduced or in an oxidative stress state, NO can induce redox reactions, leading to cell damage [45]. Our study found that SO treatment obviously reduced the secretions of IL-1β, IL-6, TNF-α, iNOS, NO and elevated IL-10 level in cerebral tissue or supernatant of LPS-induced co-culture of neuron and microglia. Simultaneously, by analysis of microglia's morphology and CD206, the results indicated that SO treatment decreased the number of pro-inflammatory microglia and increased the number of anti-inflammatory microglia in dose dependent manner, showing microglia can shift from a detrimental phenotype to a protective phenotype after ischemic stroke.

In addition, several studies have revealed that nuclear factor erythroid 2-related factor 2 (Nrf2) regulates redox homeostasis and works as an anti-inflammatory in various inflammatory-degenerative disorders, such as in cancer or neurodegeneration. As confirmed in various neurological disease models both *in vivo* and *in vitro*, the Keap1-Nrf2-ARE pathway exerts neuroprotective effects by increasing astrocytes’ glutathione secretion [46]. Relevant to chemoprevention and its related therapeutics, experimental models suggest that redox active compounds, such as natural compounds, which have been shown to act *via* hormetic dose responses, are endowed with powerful anti-inflammatory and anti-oxidant stress damage effects, displaying endpoints of biomedical and clinical relevance [45-47]. This study found that SO can reduce the expression and secretion of iNOS and NO *in vitro*, indicating that SO has the potential to resist oxidative stress. Further revealing Nrf2 mediated antioxidant stress and neuroprotective effects of SO after cerebral ischemic injury will be a promising prospect.

## CONCLUSION

In summary, our study shows that SO treatment can reverse pro-inflammatory status of microglia to decrease neuronal apoptosis and pyroptosis, which contributes to improvements of neurological function and infarction volume post ischemic stroke. The neuroprotective mechanisms are associated with regulations of TLR4/NF-κB/NLRP3/ caspase-1 and caspase-3 signals, as well as Bcl-2/Bax ratio, suggesting SO is a novel neuroinflammatory regulator in ischemic stroke.

## Data Availability

The datasets used to support the findings of this study are available from the corresponding author upon request.

## LIST OF ABBREVIATIONS

CCA: Common Carotid Artery
CNS: Central Nervous System
GSDMD: Gasdermin D
I/R: Ischemia/Reperfusion
IL-1β: Interleukin-1β
iNOS: Inducible Nitric Oxide Synthase
LPS: Lipopolysaccharide
MCA: Middle Cerebral Artery
mNSS: Modified Neurological Severity Scores
NF‐κB: Nuclear Transcription Factor Kappa B
NLRP3: Nucleotide Binding Domain Like Receptor 3
NMR: Nuclear Magnetic Resonance
NO: Nitric Oxide
RA: Rheumatoid Arthritis
ROS: Reactive Oxygen Species
SO: *Siegesbeckia orientalis* L.
TLR4: Toll-like Receptor 4
TNF-α: Tumor Necrosis Factor-alpha
TTC: 2,3,5-Triphenyltetrazolium Chloride

## References

[1] Saini V., Guada L., Yavagal D.R. (2021). Global epidemiology of stroke and access to acute Ischemic Stroke Interventions.. Neurology.

[2] Endres M., Moro M.A., Nolte C.H., Dames C., Buckwalter M.S., Meisel A. (2022). Immune pathways in etiology, acute phase, and chronic sequelae of Ischemic Stroke.. Circ. Res..

[3] Qin C., Zhou L.Q., Ma X.T., Hu Z.W., Yang S., Chen M., Bosco D.B., Wu L.J., Tian D.S. (2019). Dual functions of microglia in ischemic stroke.. Neurosci. Bull..

[4] Luo L., Liu M., Fan Y., Zhang J., Liu L., Li Y., Zhang Q., Xie H., Jiang C., Wu J., Xiao X., Wu Y. (2022). Intermittent theta-burst stimulation improves motor function by inhibiting neuronal pyroptosis and regulating microglial polarization via TLR4/NFκB/] NLRP3 signaling pathway in cerebral ischemic mice.. J. Neuroinflammation.

[5] Var S.R., Shetty A.V., Grande A.W., Low W.C., Cheeran M.C. (2021). Microglia and macrophages in neuroprotection, neurogenesis, and emerging therapies for stroke.. Cells.

[6] Anttila J.E., Whitaker K.W., Wires E.S., Harvey B.K., Airavaara M. (2017). Role of microglia in ischemic focal stroke and recovery: focus on Toll-like receptors.. Prog. Neuropsychopharmacol. Biol. Psych..

[7] Parada E., Casas A.I., Palomino-Antolin A., Gómez-Rangel V., Rubio-Navarro A., Farré-Alins V., Narros-Fernandez P., Guerrero-Hue M., Moreno J.A., Rosa J.M., Roda J.M., Hernández-García B.J., Egea J. (2019). Early toll‐like receptor 4 blockade reduces ROS and inflammation triggered by microglial pro-inflammatory phenotype in rodent and human brain ischaemia models.. Br. J. Pharmacol..

[8] Pan N., Lu L., Li M., Wang G., Sun F., Sun H., Wen X., Cheng J., Chen J., Pang J., Liu J., Guan Y., Zhao L., Chen W., Wang G. (2017). Xyloketal B alleviates cerebral infarction and neurologic deficits in a mouse stroke model by suppressing the ROS/TLR4/] NF-κB inflammatory signaling pathway.. Acta Pharmacol. Sin..

[9] Cai Q., Zhao C., Xu Y., Lin H., Jia B., Huang B., Lin S., Chen D., Jia P., Wang M., Lin W., Zhang L., Chu J., Peng J. (2024). Qingda granule alleviates cerebral ischemia/reperfusion injury by inhibiting TLR4/NF-κB/NLRP3 signaling in microglia.. J. Ethnopharmacol..

[10] Wang N., Liu Y., Jia C., Gao C., Zheng T., Wu M., Zhang Q., Zhao X., Li Z., Chen J., Wu C. (2021). Machine learning enables discovery of Gentianine targeting TLR4/NF-κB pathway to repair ischemic stroke injury.. Pharmacol. Res..

[11] Alishahi M., Farzaneh M., Ghaedrahmati F., Nejabatdoust A., Sarkaki A., Khoshnam S.E. (2019). NLRP3 inflammasome in ischemic stroke: As possible therapeutic target.. Int. J. Stroke.

[12] Xu Q., Zhao B., Ye Y., Li Y., Zhang Y., Xiong X., Gu L. (2021). Relevant mediators involved in and therapies targeting the inflammatory response induced by activation of the NLRP3 inflammasome in ischemic stroke.. J. Neuroinflammation.

[13] Sun Y., Ma J., Li D., Li P., Zhou X., Li Y., He Z., Qin L., Liang L., Luo X. (2019). Interleukin-10 inhibits interleukin-1β production and inflammasome activation of microglia in epileptic seizures.. J. Neuroinflammation.

[14] Liu Z., Hermann D.M., Dzyubenko E., Cao G., Cao X. (2023). Editorial: Modulating microglia to enhance neuroplasticity for restoring brain function after stroke.. Front. Cell. Neurosci..

[15] Tuo Q., Zhang S., Lei P. (2022). Mechanisms of neuronal cell death in ischemic stroke and their therapeutic implications.. Med. Res. Rev..

[16] Li Z., Xiao G., Wang H., He S., Zhu Y. (2021). A preparation of Ginkgo biloba L. leaves extract inhibits the apoptosis of hippocampal neurons in post-stroke mice via regulating the expression of Bax/Bcl-2 and Caspase-3.. J. Ethnopharmacol..

[17] Xian M., Cai J., Zheng K., Liu Q., Liu Y., Lin H., Liang S., Wang S. (2021). Aloe-emodin prevents nerve injury and neuroinflammation caused by ischemic stroke via the PI3K/AKT/mTOR and NF-κB pathway.. Food Funct..

[18] Puig B., Brenna S., Magnus T. (2018). Molecular communication of a dying neuron in stroke.. Int. J. Mol. Sci..

[19] Mao R., Zong N., Hu Y., Chen Y., Xu Y. (2022). Neuronal death mechanisms and therapeutic strategy in ischemic stroke.. Neurosci. Bull..

[20] Xiao B., Li J., Qiao Z., Yang S., Kwan H.Y., Jiang T., Zhang M., Xia Q., Liu Z., Su T. (2023). Therapeutic effects of Siegesbeckia orientalis L. and its active compound luteolin in Rheumatoid arthritis: network pharmacology, molecular docking and experimental validation.. J. Ethnopharmacol..

[21] Guo H., Zhang Y., Cheng B.C., Fu X., Zhu P., Chen J., Chan Y., Yin C., Wang Y., Hossen M., Amin A., Tse A.K., Yu Z. (2018). An ethanolic extract of the aerial part of Siegesbeckia orientalis L. inhibits the production of inflammatory mediators regulated by AP-1, NF-κB and IRF3 in LPS-stimulated RAW 264.7 cells.. Biosci. Trends.

[22] Nguyen T.D., Thuong P.T., Hwang I.H., Hoang T.K.H., Nguyen M.K., Nguyen H.A., Na M. (2017). Anti-hyperuricemic, anti-inflammatory and analgesic effects of Siegesbeckia orientalis L. Resulting from the fraction with high phenolic content.. BMC Complement. Altern. Med..

[23] Chu J.M.T., Xiong W., Linghu K.G., Liu Y., Zhang Y., Zhao G.D., Irwin M.G., Wong G.T.C., Yu H. (2018). Siegesbeckia orientalis l. extract attenuates postoperative cognitive dysfunction, systemic inflammation, and neuroinflammation.. Exp. Neurobiol..

[24] Linghu K.G., Xiong S.H., Zhao G.D., Zhang T., Xiong W., Zhao M., Shen X.C., Xu W., Bian Z., Wang Y., Yu H. (2020). Sigesbeckia orientalis L. extract alleviated the collagen type ii–induced arthritis through inhibiting multi-target-mediated synovial hyperplasia and inflammation.. Front. Pharmacol..

[25] Chu J.M.T., Abulimiti A., Wong B.S.H., Zhao G.D., Xiong S.H., Zhao M.M., Wang Y., Chen Y., Wang J., Zhang Y., Chang R.C.C., Yu H., Wong G.T.C. (2022). Sigesbeckia orientalis l. derived active fraction ameliorates perioperative neurocognitive disorders through alleviating hippocampal neuroinflammation.. Front. Pharmacol..

[26] Zhou Z., Zhang Y., Han F., Chen Z., Zheng Y. (2023). Umbelliferone protects against cerebral ischemic injury through selective autophagy of mitochondria.. Neurochem. Int..

[27] Han Q.Y., Zhang H., Zhang X., He D.S., Wang S.W., Cao X., Dai Y.T., Xu Y., Han L.J. (2019). DL‐3‐n‐butylphthalide preserves white matter integrity and alleviates cognitive impairment in mice with chronic cerebral hypoperfusion.. CNS Neurosci. Ther..

[28] Maniskas M.E., Roberts J.M., Trueman R., Learoyd A.E., Gorman A., Fraser J.F., Bix G.J. (2018). Intra-arterial nitroglycerin as directed acute treatment in experimental ischemic stroke.. J. Neurointerv. Surg..

[29] Alamri F.F., Shoyaib A.A., Biggers A., Jayaraman S., Guindon J., Karamyan V.T. (2018). Applicability of the grip strength and automated von Frey tactile sensitivity tests in the mouse photothrombotic model of stroke.. Behav. Brain Res..

[30] Liu P., Li H., Dong M., Gu X., Xu S., Xia S., Bao X., Xu Y., Cao X. (2023). Infiltrating myeloid cell-derived properdin markedly promotes microglia-mediated neuroinflammation after ischemic stroke.. J. Neuroinflammation.

[31] Zhang Z., Qin P., Deng Y., Ma Z., Guo H., Guo H., Hou Y., Wang S., Zou W., Sun Y., Ma Y., Hou W. (2018). The novel estrogenic receptor GPR30 alleviates ischemic injury by inhibiting TLR4-mediated microglial inflammation.. J. Neuroinflammation.

[32] Yang C., Gong S., Chen X., Wang M., Zhang L., Zhang L., Hu C. (2021). Analgecine regulates microglia polarization in ischemic stroke by inhibiting NF-κB through the TLR4 MyD88 pathway.. Int. Immunopharmacol..

[33] Ling Y., Jin L., Ma Q., Huang Y., Yang Q., Chen M., Shou Q. (2021). Salvianolic acid A alleviated inflammatory response mediated by microglia through inhibiting the activation of TLR2/4 in acute cerebral ischemia-reperfusion.. Phytomedicine.

[34] Liu M., Xu Z., Wang L., Zhang L., Liu Y., Cao J., Fu Q., Liu Y., Li H., Lou J., Hou W., Mi W., Ma Y. (2020). Cottonseed oil alleviates ischemic stroke injury by inhibiting the inflammatory activation of microglia and astrocyte.. J. Neuroinflammation.

[35] Hua F., Tang H., Wang J., Prunty M.C., Hua X., Sayeed I., Stein D.G. (2015). TAK-242, an antagonist for Toll-like receptor 4, protects against acute cerebral ischemia/reperfusion injury in mice.. J. Cereb. Blood Flow Metab..

[36] Chen W.F., Shih Y.H., Liu H.C., Cheng C.I., Chang C.I., Chen C.Y., Lin I.P., Lin M.Y., Lee C.H. (2022). 6-methoxyflavone suppresses neuroinflammation in lipopolysaccharide- stimulated microglia through the inhibition of TLR4/MyD88/p38 MAPK/NF-κB dependent pathways and the activation of HO-1/NQO-1 signaling.. Phytomedicine.

[37] Gao L., Dong Q., Song Z., Shen F., Shi J., Li Y. (2017). NLRP3 inflammasome: A promising target in ischemic stroke.. Inflamm. Res..

[38] Hu J., Zeng C., Wei J., Duan F., Liu S., Zhao Y., Tan H. (2020). The combination of Panax ginseng and Angelica sinensis alleviates ischemia brain injury by suppressing NLRP3 inflammasome activation and microglial pyroptosis.. Phytomedicine.

[39] Xu P., Hong Y., Xie Y., Yuan K., Li J., Sun R., Zhang X., Shi X., Li R., Wu J., Liu X., Hu W., Sun W. (2021). TREM-1 exacerbates neuroinflammatory injury via NLRP3 inflammasome-mediated pyroptosis in experimental subarachnoid hemorrhage.. Transl. Stroke Res..

[40] Ran Y., Su W., Gao F., Ding Z., Yang S., Ye L., Chen X., Tian G., Xi J., Liu Z. (2021). Curcumin ameliorates white matter injury after ischemic stroke by inhibiting microglia/macrophage pyroptosis through NF‐κ B suppression and NLRP3 inflammasome inhibition.. Oxid. Med. Cell. Longev..

[41] Li Q., Dai Z., Cao Y., Wang L. (2019). Caspase-1 inhibition mediates neuroprotection in experimental stroke by polarizing M2 microglia/] macrophage and suppressing NF-κB activation.. Biochem. Biophys. Res. Commun..

[42] Qi J.P., Wu A.P., Wang D.S., Wang L.F., Li S.X., Xu F.L. (2004). Correlation between neuronal injury and Caspase-3 after focal ischemia in human hippocampus.. Chin. Med. J. (Engl.).

[43] Bi W., Bao K., Zhou X., Deng Y., Li X., Zhang J., Lan X., Zhao J., Lu D., Xu Y., Cen Y., Cao R., Xu M., Zhong W., Zhu L. (2023). PSMC5 regulates microglial polarization and activation in LPS-induced cognitive deficits and motor impairments by interacting with TLR4.. J. Neuroinflammation.

[44] Xu S.Y., Jia J.Q., Sun M., Bao X.Y., Xia S.N., Shu S., Liu P., Ji S., Ye L., Cao X., Xu Y. (2023). QHRD106 ameliorates ischemic stroke injury as a long-acting tissue kallikrein preparation.. iScience.

[45] Calabrese V., Mancuso C., Calvani M., Rizzarelli E., Butterfield D.A., Giuffrida Stella A.M. (2007). Nitric oxide in the central nervous system: neuroprotection versus neurotoxicity.. Nat. Rev. Neurosci..

[46] Calabrese V., Cornelius C., Dinkova-Kostova A.T., Calabrese E.J., Mattson M.P. (2010). Cellular stress responses, the hormesis paradigm, and vitagenes: Novel targets for therapeutic intervention in neurodegenerative disorders.. Antioxid. Redox Signal..

[47] Di Rosa G., Brunetti G., Scuto M., Trovato Salinaro A., Calabrese E.J., Crea R., Schmitz-Linneweber C., Calabrese V., Saul N. (2020). Healthspan enhancement by olive polyphenols in C. elegans wild type and Parkinson’s Models.. Int. J. Mol. Sci..

