# Supplementary material for: The Extract of Siegesbeckia orientalis L. Reverses Pro-inflammatory Status of Microglia for Neuroprotection Following Ischemic Stroke in Mice
Source: Curr Neuropharmacol. 2025 Apr 25;23(13):1753–69. doi: 10.2174/011570159X349127241214045611 (PMC12645136; doi:10.2174/011570159X349127241214045611)
Supplement: Supplementary file 1 [file CN-23-13-1753_SD1.pdf]

Supplementary Material

The Extract of *Siegesbeckia orientalis* L. Reverses Pro-inflammatory Status of Microglia for Neuroprotection Following Ischemic Stroke in Mice

Liu Bowen[1,2,#], Wan Bingjie[1,#], Zhao Xinyue[1], Zhao Yonghua[3], Yu Hua[3], Jiang Xuhong[1,*], Zheng Yanrong[1,*] and Xu Yun[4,5,*]

[1]School of Pharmaceutical Sciences, The First Affiliated Hospital of Zhejiang Chinese Medical University (Zhejiang Provincial Hospital of Chinese Medicine), Zhejiang Chinese Medical University, Hangzhou, China; [2]Department of Neurology, Suzhou Hospital, Xiyuan Hospital of China Academy of Chinese Medical Sciences (Suzhou TCM Hospital Affiliated to Nanjing University of Chinese Medicine), Suzhou, China; [3]State Key Laboratory of Quality Research in Chinese Medicine, Institute of Chinese Medical Sciences, University of Macau, Macao SAR 999078, China; [4]Department of Neurology, Nanjing Drum Tower Hospital, Affiliated Hospital of Medical School, Nanjing University, Nanjing, China; [5]State Key Laboratory of Pharmaceutical Biotechnology and Institute of Translational Medicine for Brain Critical Diseases, Nanjing University, Nanjing, China

1875-6190/25 $65.00+.00 **© 2025 Bentham Science Publishers**

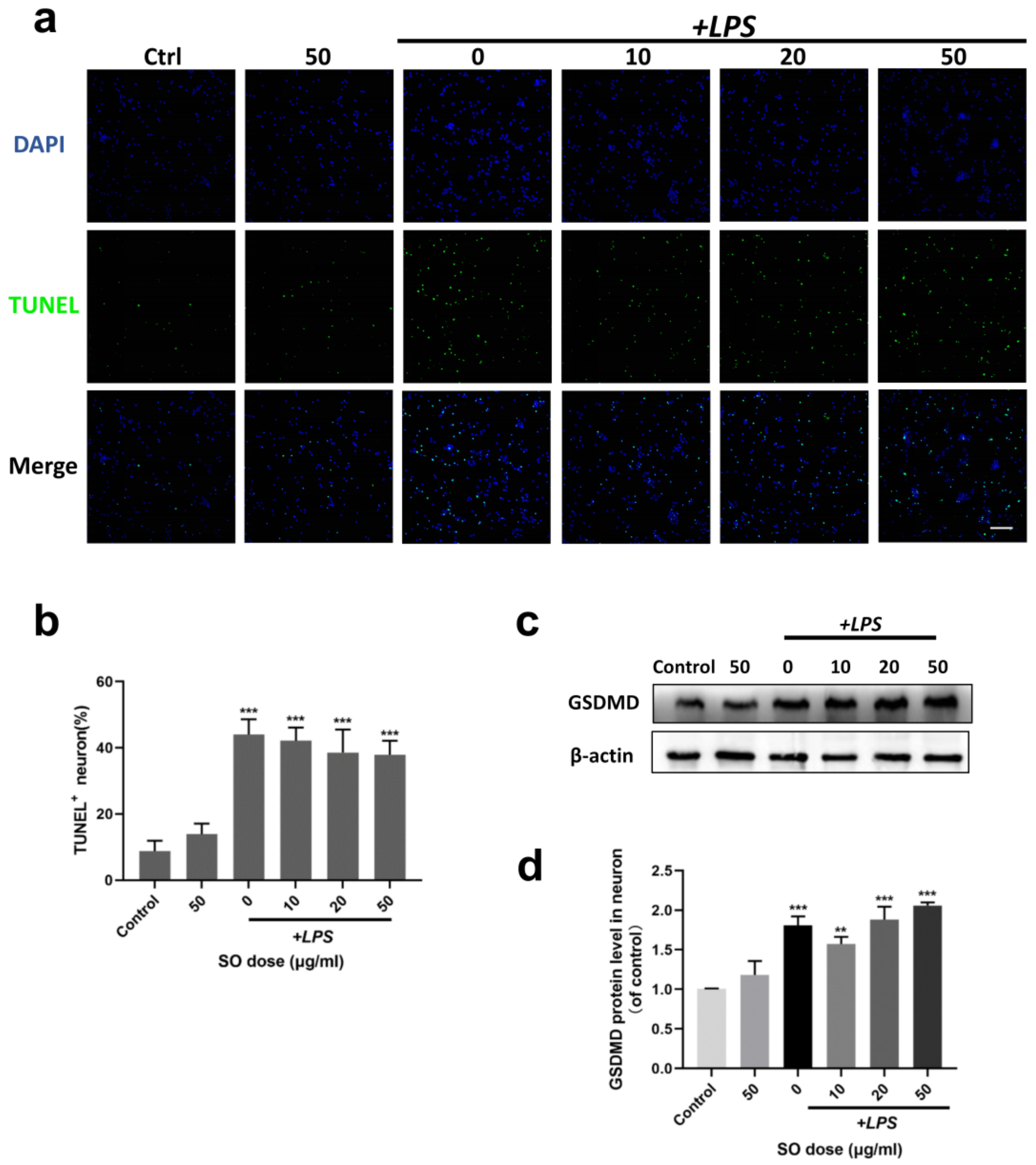

Fig. (S1). SO treatment cannot improve apoptosis and pyroptosis of primary neurons stimulated by LPS. **(a)** Cell nucleus (DAPI, blue) and TUNEL (green) are co-stained in primary neurons. **(b)** Statistics on the proportion of $TUNEL^+$ neuron expression marked as apoptotic cells. **(c)** Representative western blot bands of GSDMD expression in neurons. **(d)** Gray value quantitative statistics of GSDMD. Data are means ± SD. $^{**}p< 0.01$, $^{***}p< 0.001$ vs. Control. Scale bar= 100μm.
